# Genetic Predispositions of Glucocorticoid Resistance and Therapeutic Outcomes in Polymyalgia Rheumatica and Giant Cell Arteritis

**DOI:** 10.3390/jcm8050582

**Published:** 2019-04-27

**Authors:** Tomas Smutny, Ivan Barvik, Tomas Veleta, Petr Pavek, Tomas Soukup

**Affiliations:** 1 Department of Pharmacology and Toxicology, Centre for Drug Development, Faculty of Pharmacy in Hradec Kralove, Charles University, 500 05 Hradec Kralove, Czech Republic; smutt6aa@faf.cuni.cz (T.S.); pavek@faf.cuni.cz (P.P.); 2 Institute of Physics, Faculty of Mathematics and Physics, Charles University, 121 16 Prague, Czech Republic; ibarvik@karlov.mff.cuni.cz; 3 Department of Emergency Medicine, University Hospital in Hradec Kralove, 500 05 Hradec Kralove, Czech Republic; tomas.veleta@fnhk.cz; 4 Division of Rheumatology, 2nd Department of Internal Medicine–Gastroenterology, Faculty of Medicine in Hradec Kralove, Charles University and University Hospital in Hradec Kralove, 500 05 Hradec Kralove, Czech Republic

**Keywords:** pharmacogenetics, glucocorticoid resistance, giant cell arteritis, polymyalgia rheumatica

## Abstract

Polymyalgia rheumatica (PMR) and giant cell arteritis (GCA) are closely related chronic inflammatory diseases. Glucocorticoids (GCs) are first-choice drugs for PMR and GCA, although some patients show poor responsiveness to the initial GC regimen or experience flares after GC tapering. To date, no valid biomarkers have been found to predict which patients are at most risk for developing GC resistance. In this review, we summarize PMR- and GCA-related gene polymorphisms and we associate these gene variants with GC resistance and therapeutic outcomes. A limited number of GC resistance associated-polymorphisms have been published so far, mostly related to HLA-DRB1*04 allele. Other genes such *ICAM-1*, *TLR4* and *9*, *VEGF*, and *INFG* may play a role, although discrepancies are often found among different populations. We conclude that more studies are required to identify reliable biomarkers of GC resistance. Such biomarkers could help distinguish non-responders from responders to GC treatment, with concomitant consequences for therapeutic strategy.

## 1. Introduction

Controversy remains as to whether polymyalgia rheumatica (PMR) and giant cell arteritis (GCA) are two different pathological conditions or manifestations of a single disease. They often overlap in the same patient and affect the same population. PMR is characterized by severe, usually bilateral pain involving the musculoskeletal structures of the neck, shoulder, and/or pelvic girdles, accompanied with morning stiffness. GCA (temporal arteritis) involves large- and medium-sized blood vessel systemic vasculitis manifested by the granulomatous inflammation of the aorta and its branches. The most serious symptom of GCA is visual loss caused by vasculitis-induced ischemia [1].

Based on previous observations, about 15% of PMR patients develop GCA [2]. Recently, positron emission tomography (PET) has opened new perspectives in diagnosis for GCA and may change ranges of PMR and GCA diagnoses. In accordance with PMR/GCA overlapping, using PET scans showed that a one-third of apparently isolated PMR patients suffer from inflammatory vessel involvement or clinically unrecognized GCA [3,4,5].

Both PMR and GCA only share common clinical characteristics such as an increase in acute phase reactants reflecting systemic inflammation as well as responsiveness to corticosteroids [6].

### 1.1. Etiology and Pathogenesis

The precise cause underlying PMR and GCA is unknown, although environmental, epigenetic and genetic factors appear to play a role in the development of pathological autoimmunity.

Cytokine-mediated gene regulation is implicated in the initiation of the inflammatory process in PMR and GCA. 

In PMR, increased interstitial concentrations of pro-inflammatory cytokines have been found in affected muscles. Additionally, the inflammatory infiltrate was detected in synovial membranes predominantly in shoulders [7].

In GCA, cytokines increase the expression of endothelial cell adhesion molecules, leading to consecutive migration of leukocytes into tissue [8,9]. Subsequently, intimal hyperplasia and the fragmentation of internal elastic laminae lead to luminal narrowing. This is followed by ischemia (typically in nervus opticus), which results in visual symptoms [10].

Interestingly, ischemic complications have been associated with lower tissue expression of IL-6 and its circulating levels in GCA patients when compared with those GCA patients with no ischemic manifestations. A protective role of IL-6 can be explained by its direct effect on vascular wall components, resulting in a compensation for ischemia in GCA patients [11]. Blindness as a result of ischemic complications represents an important issue to be addressed by clinicians treating GCA patients. The identification of gene polymorphisms which may predict the risk of visual manifestations continues to be an issue of research interest. 

Some shared immunologic abnormalities between PMR and GCA include the elevation of IL-6 and IL-10 along with a similar distribution of circulating CD4+ T cell subsets such as an increase in Th17 cells and decrease in Treg cells [12,13,14].

### 1.2. Glucocorticoid Treatment of Polymyalgia Rheumatica and Giant Cell Arteritis

Treatment with glucocorticoids (GCs) is the first-line therapy for both PMR and GCA. There are wide variations in the GC treatment of PMR and GCA with respect to dosages, tapering strategies, and the duration of treatment [15,16]. GC side effects are frequently observed, thus posing further challenges. 

### 1.3. Relapsing Diseases

Approximately half of PMR patients experience a flare of disease activity upon GC tapering or discontinuation [17,18,19]. In a number of studies, increased levels of inflammatory parameters such as C-reactive protein (CRP) and erythrocyte sedimentation rate (ESR) at the beginning of the disease have been shown as risk factors for relapse/recurrence and thus longer GC therapy was required [20,21,22,23,24].

On the contrary, some published findings have described no clinical or laboratory parameters significantly associated with relapses or duration of GC therapy [25,26,27]. Recently, using a multivariate analysis, it was revealed that when patients were treated with an initial dose of 12.5 mg/day prednisolone the only factor predicting good GC responders among PMR patients is lower weight (responders 67.4 kg vs. non-responders 78.5 kg) [28]. 

Regarding GCA, Restuccia et al. [29] studied relapses in 157 patients in northern Italy. Fifty-seven patients (36.5%) experienced ≥1 flares, with 51 (46.4%) of the 110 total flares (88 relapses and 22 recurrences) appearing during the first 2 years after diagnosis. Fever and severe inflammation shown by a temporal artery biopsy seem to predict the incidence of disease flares. 

## 2. Glucocorticoid Resistance

Some patients show poor or absent response to GC treatment, with GCs having to be administered in high doses in these subjects. GC-resistant patients were defined as those who responded poorly to the initial GC regimen, or those who responded to the initial regimen but experienced a flare upon GC tapering to the maintenance dose. In this case, a flare was defined as the exacerbation or reappearance of symptoms associated with the acute phase response [30]. 

The GC resistance has been associated with a number of serious complications, including metabolic diseases as well as an increased risk of cardiovascular diseases [31,32,33,34].

Various therapeutic approaches have been applied with the aim of overcoming GC resistance. Methotrexate added to GC treatment has been associated with lower relapse rate and lower cumulative GC dosage in both PMR and GCA. Other conventional disease modifying antirheumatic drugs and TNF-α inhibitors did not seem to have a GC-sparing effect as systematically reviewed elsewhere [35]. Recent clinical trials demonstrated benefit of adjunctive tocilizumab, inhibitor IL-6R-mediated signaling, in the treatment of GCA patients [36].

**Figure 1:**
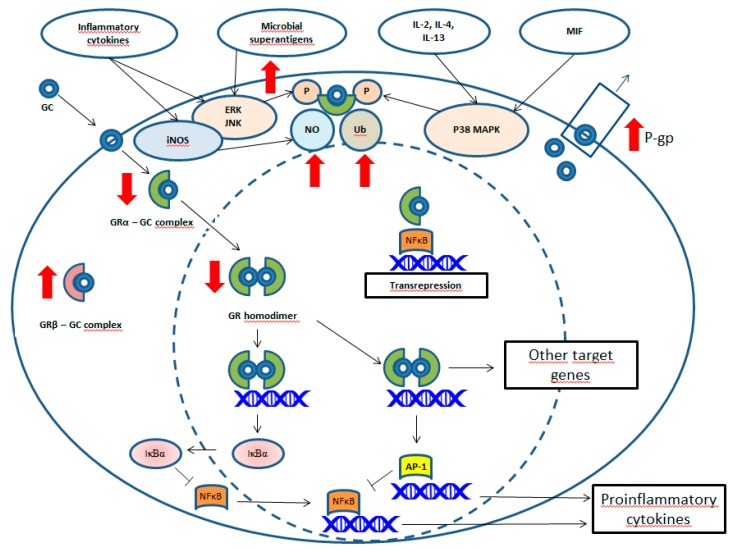
Molecular mechanisms of glucocorticoid (GC) action and GC resistance. GCs enter the target cell by passive transport through the cell membrane and they bind to the intracellular glucocorticoid receptor (GR)-alpha. GC binding to GR-beta is ineffective. Low GR-alpha: GR-beta ratio results in GC resistance. GC action is dependent on the GR-mediated transcriptional regulation of specific target genes (i.e., AP-1 gene). Their products in turn inhibit the promoter region of genes (i.e., gene encoding nuclear factor kappa B (NFκB)) which are potent transcription factors for many pro-inflammatory cytokines and adhesion molecules. Central to the anti-inflammatory action of GCs is the induction of inhibitor kappa B alpha (IκBα), which inhibits NFκB by sequestering it in the cytoplasm. The decreased cytoplasmic GC concentration is a consequence of the overexpression of P-glycoprotein (P-gp), which leads to increased P-gp-mediated efflux of GCs. The following kinases can phosphorylate (P) GR: p38 mitogen-activated protein kinase (MAPK), which is activated by IL-2, IL-4, IL-13, or macrophage migration inhibitory factor (MIF); c-Jun N-terminal kinase (JNK); and extracellular signal-regulated kinases (ERK). Nitric oxide (NO) can nitrate tyrosine residues on GR. GR can also be ubiquitinated (Ub), which results in the degradation of GR by the proteasome. Red arrows indicate increase or decrease in GC resistance.

The molecular basis of GC resistance (see Table 1, Figure 1) is poorly understood in PMR and GCA patients, however it has been widely studied in other inflammatory conditions. Immune system abnormalities have a significant role in disease pathogenesis and are able to dictate, not only clinical features, but also treatment outcomes. GC resistance may occur as a result of prolonged exposure to inflammatory cytokines [37]. Elevated expression of pro-inflammatory cytokines such as IL-1β, TNFα and IL-6 in temporal biopsies reflects the stronger systemic inflammatory reaction in GCA patients. Those patients with higher tissue TNFα production also had longer requirements for prednisone administration [38]. Several studies showed that persistently elevated levels of serum IL-6 during GC therapy were significantly associated with an increased risk of relapse and recurrence of PMR activity, as well as with a prolonged course featuring an increased need for prednisolone [17,20]. 

Glucocorticoid receptor (GR) is the target molecule for GC treatment. The success of GC therapy seems to reflect GR staining in the chronic inflammatory cells in GCA. Patients highly responding to GCs tend to have more GR-positive lymphocytes/monocytes comparing to those with incomplete treatment response. The number CD68-positive cells, a hallmark of the chronic inflammation, were also related to treatment success and GC resistance [39].

Braun et al. [39] aimed to determine the value of clinical genetic findings as well as the expression of GR for discriminating between GCA and PMR patients who achieve either complete remission or partial remission after GCs, as opposed to those who are resistant to GC treatment. A study on GC receptor polymorphisms revealed that more than 90% of patients had the wild-type genotype (homozygote) of R23K and N363S polymorphisms; no evidence was found that these polymorphisms influenced response to treatment with GCs. In our literature search, we found no other studies dealing with GC resistance and GR-related polymorphisms in the treatment of PMR and GCA.

## 3. Methods

The systematic search for genetic associations to GCA and PMR was performed using DisGeNET database. DisGeNET version 5.1 (released in May, 2018) contains a compilation of genes associated to diseases integrating several widely used gene-disease databases [40]. Search results (54 for PMR and 240 for GCA) were further verified in full text papers available on PubMed. Duplicated findings and incorrectly categorized articles (as those including GCA for gastric cardia adenocarcinoma instead of giant cell arteritis) were excluded. Evidence relevant to the effect of GC treatment and ischemic complications (the prognostic factor for a worse GC treatment response) were included in review.

Additionally, two reviewers independently searched PubMed database for relevant publications without restriction of article type using “polymyalgia rheumatica” or “giant cell arteritis” as key words. 

## 4. Genetic Studies of GCA and PMR with Relevance of GC Treatment Outcome

We performed an overview of genetic predisposition to GC treatment outcomes in GCA (Table 2) and PMR (Table 3) as a basis for further genetic studies, as well as for further consideration of new therapeutic strategies. The genes included in the review play a role in the pathogenesis of GCA and PMR, and they may also be involved in GC resistance and thus in disease severity.

Reports included in this manuscript encompass different approaches to defining GC resistance from a clinical point of view. The phenomenon of GC resistance in PMR and GCA is generally manifested by the absence of an expected response to a recommended GC dosage. GC resistance has been defined as experience of relapse/recurrence and/or higher and prolonged GC requirements in PMR patients. The persistence of various ischemic manifestations following GC treatment was considered as a marker of GC resistance in GCA patients. Since no universal definition of GC resistance could be obtained, we stated details on GC resistance for each study into Table 2 and Table 3. 

### 4.1. Human Leukocyte Antigen (HLA)

Rauzy et al. observed a significantly higher frequency of HLA-DRB1*04 in a group of GCA patients studied over two years. An association of GCA with HLA-DRB1*04 seemed to accompany GC resistance [41]. According to Dababneh et al., HLA-DRB1*04 was particularly pronounced in those Spanish patients with severe visual complications [42]. This supports the idea proposed by Rauzy et al. [43] that HLA-DRB1*04 may be a genetic marker of severity in GCA. In contrast, although Combe et al. described an increased frequency of HLA-DRB1*04 in their case series of 42 French patients with GCA, they did not find any significant relationship between the HLA-DRB1 gene and markers of disease severity and activity. These discrepancies may be related to the different ethnic backgrounds of the investigated groups.

HLA-DR4 is associated with rheumatoid arthritis (RA), in particular with the prognostically more severe form (early onset RA). Rheumatoid factor seronegative elderly onset RA and isolated PMR patients do not seem to be associated with DRB1*04 or *01. Instead, both pathological states appear to have a similar genetic basis (disease susceptibility) such as association with DRB1*13/14 [75]. Salvarani et al. studied the risk factors associated with relapse/recurrence in PMR, aiming at clinical and laboratory parameters, as well as HLA-DRB1* antigens using a multivariate analysis. This work showed that increased ESR at diagnosis (>72 mm 1st h) along with the presence of the rheumatoid epitope encoded by a non-DR4 (particularly DR1) allele are independent risk factors of relapse/recurrence. These results support the presumption that DR1 may be implicated in the prognosis of PMR patients, helping to identify those with a more severe form of the disease [66].

### 4.2. Intercellular Adhesion Molecule-1

Regarding ICAM-1 gene polymorphisms, the mutation of glycine (G) with arginine (R) at codon 241 was found to contribute to an increased risk of PMR relapse/recurrence in Italian patients. G/R polymorphism occurs in the domain 3 of ICAM-1, which is involved in the interaction with leukocyte integrins. Therefore, it was proposed that R241 mutation could confer a more effective adhesive function of ICAM-1, with a subsequent impact on the inflammatory response [72], although discordant data have been also published. Amoli et al. did not confirm ICAM-1 241 polymorphism as an independent risk factor of PMR severity [71], the potential explanation of which being that both groups studied consisted of two different populations varying in ethnic background, since Italian and Spanish donors were examined. Moreover, the association between the HLA-DRB1*0401 allele and the risk of relapse was described in Spaniards with PMR. All relapsed patients with HLA-DRB1*0401 also carried the GG genotype of ICAM-1 at codon 241 [71]. 

In the case of GCA severity, visual manifestations were not associated with polymorphism of ICAM-1 at codon 241 or codon 469 (biallelic polymorphism Lys/Glu) in patients from northwest Spain. Additionally, an association between HLA-DRB1*04 and visual complications was observed, but no interaction between HLA-DRB1*04 and ICAM-1 polymorphism could be found in the series of examined patients [49].

### 4.3. Interleukin 6

IL-6 is a significantly produced cytokine in PMR and GCA patients, which is considered as a potential marker of disease activity sensitive to GC treatment [76]. IL-6 expression is affected by promoter polymorphism at position −174 G/C in the IL-6 gene. It was found that patients with isolated PMR and homozygous for the C allele had elevated serum levels of IL-6 as compared to carriers of the GC and GG genotypes, but no differences among genotypes were reported in terms of relapse/recurrence frequency [67]. Next, the G/C 174 genotype was not associated with the clinical features of GCA demonstrated as ischemic complications [51]. As was the case with the Italian patients from the previous two studies, no associations between polymorphism at −174 G/C of IL-6 with disease severity were observed in a cohort of Spanish patients with isolated PMR nor in GCA patients [52].

### 4.4. RANTES and CC Chemokine Receptor 5

The chemokine RANTES (Regulated upon Activation, Normal T cell Expressed and presumably Secreted) activates monocytes and memory T cells, the main cell types infiltrating PMR synovial tissue via interaction with the CC chemokine receptor 5 (CCR5) [69]. The gene encoding CCR5 is polymorphic with the identified non-functional 32-bp deletion allele [77]. It has been hypothesized that polymorphism in the CCR5 gene might help explain the higher circulating levels of RANTES and the severity of disease in PMR patients. Nevertheless, according to the results obtained from the study with PMR patients of Italian origin, no significant association was observed between the CCR5 genotype and the therapeutic outcomes measured as duration of therapy, frequency of relapse/recurrence, as well as the initial and cumulative prednisone dose [69]. 

### 4.5. Monocyte Chemoattractant Protein-1

Monocyte chemoattractant protein-1 (MCP-1, CCL2) is another CC chemokine which attracts and activates monocytes and T cells [57]. MCP-1 mRNA levels were found to be elevated in temporal artery samples gained from GCA patients as compared to controls (31 ± 15.6 vs. 0.44 ± 0.1, *p* = 0.0001), with even higher levels found in patients who had suffered two or more relapses than in patients with no relapse (127 ± 82 vs. 11 ± 5.5, *p* = 0.0233). MCP-1 mRNA concentration also correlated with the cumulative prednisolone dose (*R* = 0.533, *p* = 0.0024). These findings indicate that MPC-1 may participate in the persistence of inflammation in GCA patients [78]. Further, three common single nucleotide polymorphisms (SNPs) in the MCP-1 gene were studied in the group of GCA Spanish patients, but none of the SNPs was associated with disease severity presenting as visual manifestations [57]. 

### 4.6. Interferon Gamma

Interferon gamma (IFN-γ) is a cytokine involved in the pathogenesis of only GCA but not PMR as revealed by histological analyses of the temporal arteries of both PMR patients without evidence of arteritis as well as GCA patients. This results indicate the role of this cytokine in the progression of overt arteritis [14]. Dinucleotide (CA) repeat polymorphism within the first intron of the IFN-γ gene (INFG) was assessed in association with the clinical features of Spanish patients suffering from GCA [54]. It was shown that GCA patients with visual ischemic manifestations were characterized by the presence of a lower frequency of INFG allele *4 (a low IFN-γ producer) as compared with GCA patients without visual ischemic complications. On the contrary, allele *3 (a high IFN-γ producer) was revealed in a higher frequency among GCA patients with visual ischemic manifestations, suggesting the relevant role of functional polymorphisms of the INFG in the clinical severity of GCA [54]. Later, the receptor for INF-γ was also interrogated, however, none of the three tested single nucleotide polymorphisms (−611A/G, +189G/C and +95C/T) of the IFNGR1 gene provided any evidence of linkage with clinical manifestations of GCA [53].

### 4.7. Interleukin 2 and Interleukin 21

The phenotypic expression of GCA was disclosed to be associated with SNP, referred to as rs6822844 G/T and situated in the intergenic region between IL-2 and IL-21 genes. In this regard, the increased frequency of the minor allele T occurred in patients with severe ischemic events as well as in patients experiencing jaw claudication. It has been proposed that the role of this noncoding polymorphism is involved in microRNA production, with a subsequent alteration in target gene expression [47].

### 4.8. Interleukin 1 Receptor Antagonist and Interleukin 10

Anti-inflammatory mechanisms, including the effects of receptor antagonists, e.g., the IL-1 receptor antagonist (IL-1Ra) and anti-inflammatory cytokines such as IL-10, may favor disease treatment outcomes by the delicate balancing of pro-inflammatory cytokines. 

Although IL-1Ra binds to the IL-1 receptor, it does not trigger any intracellular response. Since it has been suggested that genetic polymorphisms of IL-1Ra may result in differential in vivo protein levels, the impact of a tandem-repeat polymorphism within intron 2 of the IL1-Ra gene (IL1-RN) on the severity of PMR and GCA was investigated [56]. Interestingly, although the IL-1RN*2 allele was proposed to be responsible for higher plasma levels of IL-Ra, and although the IL-1RN*2/2 genotype is more distributed among PMR patients compared to GCA patients, no associations were found between the presence of the IL-1RN*1 or IL-1RN*2 allele and the number of relapses, duration of GC treatment, or the cumulative prednisone dose among Spanish patients with PMR or GCA [56].

Another work focused on an analysis of several polymorphisms regulating the expression of genes coding cytokines, namely IL-1A (+4845), IL-1B (−511), IL-1B (+3954), the TNFα gene (−308) and IL-1RN Intron 2. The reported results support no associations between these gene variants and disease severity in PMR patients of Italian origin [70].

IL-10 is Th2-derived cytokine which suppresses the production of pro-inflammatory Th1-derived IFN-γ. IL-10 promoter polymorphisms −592C/A and −1082A/G have been studied in terms of the prognosis of PMR and GCA patients, but no association between these polymorphisms and clinical phenotype was found [58,59,73].

### 4.9. Corticotropin-Releasing Hormone

The hypothalamic production of the corticotropin-releasing hormone (CRH) is stimulated by inflammatory cytokines like IL-1, IL-6 and TNF. In turn, CRH helps to control homeostasis during immune-meditated inflammatory stress. The Spanish study regarding the distribution of two biallelelic CRH promoter polymorphisms localized at positions 1273 (alleles A1 and A2) and 225 (alleles B1 and B2) revealed no association with the severity of disease in patients with isolated PMR. This was not the case in GCA patients from the same region (Lugo, Spain), where a higher frequency of the allele A2 was associated with the development of visual ischemic manifestations. These data, however, were obtained from a small group of GCA patients with visual complications (*n* = 14), a fact which limits the unequivocal interpretation of the results [79].

### 4.10. Toll-like Receptor 4 and 9

Toll-like receptors (TLRs) are important components of innate and acquired immune responses. Among these, TLR4 gene has been proposed as a genetic marker of clinical outcomes in patients with PMR. As assessed by an analysis of its two gene polymorphisms Asp299Gly and Thr399Ile, the Thr399Ile CC genotype was associated with the administration of a higher cumulative dose of prednisone in PMR patients as compared to healthy controls (5.6 ± 4.8 vs. 2.1 ± 0.5 g; *p* = 0.031). These findings can be explained by longer prednisone treatment (41.8 ± 39.3 vs. 16.0 ± 10.4 months; *p* = 0.10), and a higher number of relapses (0.9 ± 1.3 vs. 0.0 ± 0.0; *p* = 0.07) for Thr399Ile CC genotype. However, when the functional consequences of TLR4 variants were examined, none of these revealed a different expression on B cells, T cells or monocytes, or showed a distinct response in intracellular cytokine production using stimulated monocytes [68].

Only three studies, all carried out on Mediterranean population, have been published so far looking for the association among TLR4 gene polymorphisms and clinical features of GCA. However, reported results did not find any association of distinct polymorphisms with clinical manifestations such as ischemic symptoms or the prognosis of the disease [61,62,63]. 

As shown in another study, GCA patients carrying the TC genotype of the TLR9 gene T−1486C polymorphism needed a shorter duration of GC treatment. Next, patients with the CC genotype of the TLR9 T−1237C polymorphism suffered from an increased number of relapses/recurrences suggesting a role of TLR9 gene variants in prognosis of GCA. On the other hand, studied polymorphisms did not have any impact on therapeutic outcomes of PMR patients [64].

### 4.11. Nuclear Factor of κB1

The nuclear factor of the κ-light polypeptide gene enhancer in B cells 1 (NFκB1) is a key activator of genes implicated in immune processes. NFκB is also the main target of GC treatment [31]. Of note, NFKB1 promoter polymorphism described as the presence or absence of 4-base pair deletion (−94ins/delATTG) has been associated with altered NFκB1 expression. Nevertheless, one population study showed the unlikelihood that 94ins/delATTG NFKB1 promoter polymorphism has any impact on clinical manifestations of GCA [50]. 

### 4.12. Protein Tyrosine Phosphatase Non-Receptor 22

Protein tyrosine phosphatases together with protein tyrosine kinases tightly orchestrate signal transduction in cells, with the deregulation of cell signaling supposed as being implicated in inflammatory pathological processes. One lymphoid-specific phosphatase (Lyp) encoded by the PTPN22 (protein tyrosine phosphatase non-receptor 22) gene has been studied regarding its involvement in severe ischemic manifestations among GCA patients. PTPN22 is a polymorphic gene at position C1858T, which plays a role in T cell activity. In one Spanish study, however, no evidence was found related to any association between PTPN22 polymorphism and clinical expression of GCA [48]. In the same line, meta-analysis of four independent cohorts (911 patients in total) on two PTPN22 polymorphisms (rs2476601 and rs33996649) showed no differences among GCA patient subphenotypes. Nevertheless, when subpopulations of GCA patients were compared to healthy patients (case vs. control), minor allele (rs2476601) was significantly associated with both GCA patients suffering from visual ischemic manifestations and irreversible occlusive disease [80].

### 4.13. Vascular Endothelial Growth Factor

In patients with active PMR/GCA, elevated circulating vascular endothelial growth factor (VEGF) levels have been described. Increased VEGF serum concentrations were reduced by GC treatment [81]. VEGF represents an important pro-angiogenic factor controlling neovascularization. The role of two VEGF promoter polymorphisms were assessed regarding ischemic complications among GCA patients of Spanish origin. Interestingly, the VEGF −634 G allele was found more frequently than the C allele in GCA patients with severe ischemic manifestations as compared to in the remaining patients or controls. Moreover, a higher risk of severe ischemic complications was detected among patients carrying the 634 GG genotype; however, no association was found regarding another studied polymorphism G1154A [55]. Nevertheless, in disagreement with this Spanish study, results with Italian GCA patients with or without ischemic manifestations revealed that the distribution of VEGF G634C polymorphism showed no significant differences [82]. These disparities among results again support the role of different genetic backgrounds in severity of disease among diverse populations. 

### 4.14. Platelet Glycoprotein IIIa

Although visual manifestations in GCA are assigned to occlusive vasculopathy as a result of intimal hyperplasia, thrombotic occlusion may also be involved. Indeed, cranial ischemic complications have been associated with PlA2 allele (rs5918) of the platelet glycoprotein IIIa (GPPIIIA), a subunit of the platelet membrane receptor involved in thrombus formation. GCA patients being homozygous for PlA2 allele have been in increased risk of visual loss [65]. 

### 4.15. Interleukin 17A

IL-17A, often referred to as IL-17, is a pro-inflammatory cytokine. The IL-17-triggered release of IL-6 activates the STAT3 pathway, which further activates the NFκB pathway [83]. Marquez et al. produced evidence of an association between GCA susceptibility and the IL-17A G197A (rs2275913) and G8065A (rs7747909) SNPs. This evidence points to the role of IL-17-producing Th17 cells in this type of vasculitis [84]. Indeed, Th17 cells have been found in GCA lesions [85]. Furthermore, a higher expression of IL-17A as well as other cytokines implicated in Th17 differentiation and function has been discovered in temporal artery biopsies taken from GCA patients [38,83,86]. IL-17-producing Th17 cells have been shown to contribute to systemic and vascular symptoms of GCA, but through a different pathogenic pathway than that mediated by IFN-γ-producing Th1 cells. Moreover, it has been suggested that Th17 cells exert more important influence in the early disease process, whereas Th1 cells seem to be more involved in the chronic disease [87]. 

**Figure 2:**
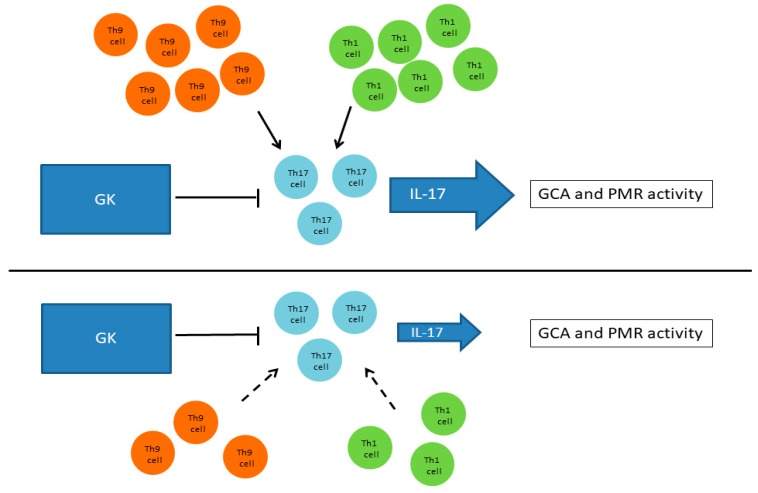
Potential involvement of Th cell subpopulations in GC resistance. Glucocorticoids effectively suppress Th17 cell type but have no effect either on Th1 cells, or on the IL-9-producing Th9 cell. This may indicate the potential implication of Th9 and Th1 cells in glucocorticoid resistance.

The study by Deng et al. showed not only the crucial role that Th17 cells have in the development of GCA, but also provided evidence that GCs effectively suppress Th17 cells [85]. Espígol-Frigolé et al. confirmed the high relevance of Th17 cells in the immunopathology of GCA, as well as in the response to GC treatment [86]. On the contrary, GCs have shown an effect neither on Th1 cells [85] nor on IL-9-producing Th9 cells [88]. These findings may indicate a potential implication of Th1 and Th9 cells in GC resistance. As stated above, Th17 cells are sensitive to GC-mediated suppression, thus the Th17/Th1 ratio may be a potential marker for the assessment of GC resistance in GCA patients (Figure 2) [85,86].

It is also worth mentioning that IL-17A can act as a pro-atherogenic factor. Pre-existing atherosclerosis has been shown as a potential risk factor for ischemic complications in GCA. Therefore, it is tempting to assume that IL-17A might also affect the severity of this type of vasculitis, thus presenting a promising therapeutic target for GCA [84]. In addition, IL-17A gene polymorphisms are promising candidate markers for the study of GC treatment outcomes in GCA [84].

### 4.16. Other Genetic Associations

Although GCA is considered complex polygenic disease, in which more polymorphic genes are supposed to affect its clinical manifestation, many studies did not succeed to find associations between clinical expression of GCA and gene polymorphisms such as ITGAM rs1143679 [89], STAT4 rs7574865 [90], IL2RA rs2104286 [91], 6q23/TNFAIP3 gene region rs6920220 [92], TRAF1/C5 region rs10818488 and rs2900180 [93], TRAF6 rs540386 [94], IL23R rs1343151, IL12RB2 rs3790567 [95], CSK rs1378942 and rs34933034 [80], CD226 rs727088, rs34794968, and rs763361 [96], MPO rs2333227 [97], C-reactive protein rs1417938, rs1800947, rs1205, and rs3093059 [98], CCR6 rs3093024 [99], IRF5 rs2004640 and 5-bp CGGGG insertion/deletion [100], BANK1 rs17266594, rs10516487, and rs3733197 [101], endothelial nitric oxide synthase (eNOS) rs1799983 and 4a/b polymorphism in intron 4 (variable number of tandem-repeats, VNTRs) [102].

Some other reports showed marginally significant associations of polymorphism either with susceptibility to visual ischemic manifestations or severe ischemic manifestations within a cohort of GCA patients. The first is exemplified by the minor allele T of CD40 (rs1883832 C/T polymorphism) [103], the latter is true for A allele of rs13277113 (A/G), which is located between C8orf13 and B-lymphoid kinase (BLK) genes [104].

### 4.17. Deregulated Cell Signaling Pathways in PMR and GCA

It is currently accepted that the cytokine axes such as IL-12/IFN-γ and IL-6/IL-17 are the most relevant pathways involved both in medium and large vessel vasculitis as well as in vasculitis associated with psoriasis [105,106,107]. In GCA and PMR, some recent observations have suggested that these pathways may lead to different disease outcomes [83]. For example, elevated Th1 response, in which increased IFN-γ levels would trigger strong macrophage activation, appears to be related to granulomatous inflammation and blindness. Nevertheless, polarization of the response to Th17 is associated with the presence of PMR, with higher relapse rates, as well as with lower patient susceptibility to developing blindness. 

## 5. Conclusions

Regarding PMR and GCA patients, our current understanding of GC resistance associated with MHC and non-MHC gene polymorphisms is relatively limited. One reason for this is that most conducted clinical studies enrolled only small patient groups with the lack of statistical power. Additionally, the primary goal of most clinical studies was aimed at disease susceptibility. The HLA-DRB1*04 allele carriage appears to be the promising prognostic factor for GC resistance at least within specific ethnic populations. Further effort should focus among others on ICAM-1, TLR4 and 9, VEGF, and INFG polymorphisms since recent preliminary findings have suggested their contribution to GC resistance. Although IL-6 is potential serum marker, but polymorphisms of IL-6 gene is not currently found as a genetic marker of PMR/GCA disease activity. The detected genetic associations will require further validation in larger populations of different ethnicity. Such approaches may result in promising therapeutic implications, since the findings could help to identify patients as potential responders or non-responders to GC treatment, with concomitant consequences in therapeutic strategies.

## Figures and Tables

**Table 1:** Mechanisms of glucocorticoid resistance.

Genetic Polymorphisms or Mutations of GR
Chrousos syndrome–primary glucocorticoid resistance Defective ligand binding to GR Lower transport GR–GC complex to nucleus Defective transactivation
**Post-translational modifications of GR**
Phosphorylation Nitrosylation Ubiquitination
**Increased GRβ expression**
**Increased activity of pro-inflammatory transcription factors and kinases**
NFκB, AP1 JNK, STAT5, JAK3
**Defective histone acetylation**
Reduced acetylation of lysine 5 on histone 4 Reduced histone deacetylase 2
**Increased oxidative stress**
Increased phosphoinositide-3-kinase-δ activation
**Increased P-glycoprotein**
Increased efflux of steroids
**Inactivation of glucocorticoids**
Expression of 11β-hydroxysteroid dehydrogenase 2

AP1, activator protein 1; JAK3, Janus kinase 3; JNK, c-Jun N-terminal kinase; NFκB, nuclear factor kappa B; STAT, signal transduction-activated transcription factor.

**Table 2:** Genetic susceptibility to glucocorticoid treatment outcomes in giant cell arteritis.

Polymorphism	Population	Allele or Genotype	Clinical Features	Allele Freq. (%)	OR (95% CI)	*p*	*R*.
HLA-DRB1	41 GCA patients French patients	*04	GC resistant vs. GC sensitive patients	88.9 vs. 37.5	RR 2.37 (1.43–3.92)	0.0064	[41]
HLA-DRB1	53 GCA patients 145 controls Spanish patients (Lugo region)	*04	patients with visual manifestations (partial or complete visual loss) vs. healthy controls	63.6 vs. 26.2	4.9 (1.2–21.4)	0.008	[42]
HLA-DRB1	42 GCA patients 79 PMR patients French patients	*04	steroid dosage in allele positive vs. all patients			NS	[43]
		*04	number of relapses in allele positive vs. all patients			NS	
HLA-DRB1	39 GCA patients Italian patients (Reggio Emilia)	*04	patients with vs. without systemic signs and/or symptoms	34.5 vs. 0	RR 1.5 (1.2–2.0)	0.04	[44]
HLA-DRB1	44 GCA patients Spanish patients (Cantabria)	*07	patients with vs. without ischemic complications	30 vs. 19	1.8 (0.7–5.1)	NS	[45]
		*01	patients with vs. without ischemic complications	10 vs. 17.2	0.5 (0.1–2.1)	NS	
		*13	patients with vs. without ischemic complications	6.7 vs. 12.1	0.5 (0.1–2.7)	NS	
HLA-DRB1	102 GCA/PMR patients Danish patients	*04	cumulative dose of prednisolone for the first year of treatment in allele positive vs. negative patients		median: 4.5g vs. 3.8g	0.011	[46]
MBL A/O		AO + OO vs. AA	cumulative dose of prednisolone for the first year of treatment in patients	46 vs. 54		NS	
IL2-IL21 rs6822844 G/T	272 GCA patients Spanish patients	T	patients with vs. without severe ischemic events *	12.8 vs. 7.7	1.72 (0.97–3.05)	0.05 0.30 ^#^	[47]
		T	patients with vs. without jaw claudication	13.7 vs. 8.2	1.76 (1.02–3.04)	0.04 0.24 ^#^	
		T	patients with vs. without permanent visual loss	17.5 vs. 10	2.09 (0.90–4.86)	0.13 0.78 ^#^	
PTPN22 rs2476601 1858C/T	96 GCA patients Spanish patients (Lugo region)	C or T	patients with vs. without severe ischemic events *			NS	[48]
ICAM-1 241 R/G	58 GCA patients Spanish patients (Lugo region)	R or G	patients with vs. without visual manifestations **			NS	[49]
ICAM-1 469 K/E		K or E				NS	
HLA-DRB1		*04		40 vs. 12.1	4.8 (1.3–18)	0.01	
NFKB1 −94ins/delATTG	96 GCA patients Spanish patients (Lugo region)	ins or del	patients with vs. without severe ischemic events *			NS	[50]
IL-6 −174 G/C	126 GCA patients Italian patients (Reggio Emilia)	G or C	patients with vs. without ischemic complications ***			NS	[51]
IL-6 −174 G/C	62 GCA patients Spanish patients (Lugo region)	G or C	patients with vs. without visual manifestations **			NS	[52]
IFNGR1 rs1327474 −611A/G	216 GCA patients Spanish patients	A or G	patients with vs. without (severe) ischemic events */**			NS	[53]
INFGR1 rs11914 +189G/C		G or C				NS	
INFGR1 rs7749390 +95C/T		C or T				NS	
IFN-γ gene dinucleotide (CA) repeat within the first intron	59 GCA patients Spanish patients (Lugo region)	*4 (128 bp)	patients with vs. without visual manifestations **	17.9 vs. 42.5	0.36 (0.13–1.00)	0.05	[54]
		*3 (126 bp)	patients with vs. without visual manifestations **	71.4 vs. 44.4	3.13 (1.27–7.68)	0.01	
VEGF rs1570360 −1154 G/A	103 GCA patients 226 controls Spanish patients (Lugo region)	G or A	patients with vs. without severe ischemic events *			NS	[55]
VEGF rs2010963 −634 G/C		G	patients with vs. without severe ischemic events *	75.5 vs. 60	2.05 (1.13–3.71)	0.017 0.034 ^#^	
		G	patients with severe ischemic events * vs. healthy controls	75.5 vs. 63.7	1.75 (1.08–2.88)	0.021 0.042 ^#^	
		GG + GC vs. CC	patients with vs. without severe ischemic events *		5.26 (1.39–19.98)	0.009 0.018 ^#^	
IL-1RN 86 bp VNTRs within the second intron	69 GCA patients 437 controls Spanish patients (Cantabria)	*1 (four repeats) or *2 (two repeats)	relapses/recurrences, number of relapses, duration of corticosteroid therapy, cumulative prednisone dose, presence of ischemic manifestations ***			NS	[56]
MCP-1 rs2857657 intron 1 (G/C)	79 GCA patients 99 controls Spanish patients (Lugo region)	G or C	patients with vs. without visual manifestations ** or controls			NS	[57]
MCP-1 rs4586 exon 2 (T/C)		T or C				NS	
MCP-1 rs13900 3′UTR (C/T)		C or T				NS	
IL-10 rs1800872 −592C/A	103 GCA patients 232 controls Spanish patients (Lugo region)	C or A	severe ischemic complications *			NS	[58]
IL-10 rs1800896 −1082G/A		G or A				NS	
IL-10 rs1800872 −592C/A	140 GCA patients 200 controls Italian patients (Reggio Emilia)	C or A	severe ischemic complications ***			NS	[59]
IL-10 rs1800896 −1082G/A		G or A				NS	
CD24 rs3838646 P1527 TG/del	120 GCA patients 195 controls Spanish patients (Lugo region)	TG or del	severe ischemic complications *			NS	[60]
CD24 rs8734 C52T		C or T				NS	
TLR4 rs4986790 +896A/G	155 GCA patients 210 controls Italian patients (Reggio Emilia)	A or G	patients with vs. without visual loss and/or cerebrovascular accidents			NS	[61]
TLR4 rs4986790 +896A/G	210 GCA patients 678 controls Spanish patients (Lugo region, Madrid, Granada)	A or G	patients with vs. without visual ischemic complications **/severe ischemic manifestations *			NS	[62]
			patients with visual ischemic complications vs. healthy controls **/severe ischemic manifestations *			NS	
TLR4 rs4986790 +896A/G	72 GCA patients 126 controls Spanish patients (Cantabria)	A or G	at least one relapse/recurrence, number of relapses, duration of GC treatment, cumulative prednisone dose; ischemic manifestations ***			NS	[63]
TLR4 rs4986791 +1196C/T		C or T				NS	
TLR9 rs187084 −1486T/C	97 GCA patients 128 controls Spanish patients	TC vs. TT	a shorter duration of GC therapy			0.029	[64]
TLR9 rs5743836 −1237T/C		CC vs. TT	a higher number of relapses/recurrences			0.045	
GPIIIa rs5918 PlA1/A2 +1565T/C	140 GCA patients 241 controls Italian patients (Reggio Emilia)	PlA2	patients with vs. without cranial ischemic complications	27.4 vs. 15.1	2.1 (1.1–4.1)	0.037	[65]

HLA, Human leukocyte antigen; ICAM-1, Intercellular adhesion molecule-1; IFN-γ, Interferon gamma; INFGR1, Interferon gamma receptor 1; IL, Interleukin; IL-1Ra, IL-1 receptor antagonist; GC, glucocorticoids; GPIIIa, Platelet glycoprotein IIIa; MBL, Mannose-binding lectin; MCP-1, Monocyte chemoattractant protein-1; NFκB1, Nuclear factor of κ-light polypeptide gene enhancer in B cells 1; PTPN22, Protein tyrosine phosphatase non-receptor 22; TLR, Toll-like receptor; VEGF, Vascular endothelial growth factor. * At least one of the following complications: visual manifestations, cerebrovascular accidents, limb claudication, jaw claudication. ** At least one of the following complications: permanent visual loss, amaurosis fugax, diplopia. *** At least one of the following complications: visual loss, jaw claudication, cerebrovascular accidents, aortic arch syndrome. NS—no statistical differences. ^#^ values corrected by the number of comparisons.

**Table 3:** Genetic susceptibility to glucocorticoid treatment outcomes in polymyalgia rheumatica.

Polymorphism	Population	Allele or Genotype	Clinical Features	Allele Freq. (%)	OR (95% CI)	*p*	*R*.
HLA-DRB1	89 PMR patients Spanish patients (Cantabria region)	*09	patients with vs. without relapses	5.6 vs. 0		0.04	[45]
		*0101	patients with vs. without relapses	11.1 vs. 8.3	1.4 (0.4–5.2)	0.07	
		*07	patients with vs. without relapses	11.1 vs. 4.2	2.9 (0.6–13.6)	0.2	
HLA-DRB1	91 PMR patients Italian patients (Reggio Emilia)	*01	patients with vs. without relapses/recurrences		RH 1.56 (0.54–4.5)	0.01	[66]
		*10	patients with vs. without relapses/recurrences		RH 2.63 (0.23–30.11)	0.005	
IL-6 −174 G/C	84 iPMR patients Spanish patients (Lugo region)	G or C	patients with vs. without relapses/recurrences			NS	[52]
IL-6 −174 G/C	112 iPMR patients Italian patients (Reggio Emilia)	G or C	patients with vs. without relapses/recurrences			NS	[67]
TLR4 rs4986791 +1196 C/T	164 PMR patients Spanish patients	CC	cumulative dose of prednisone in PMR patients		5.6 ± 4.8 vs. 2.1 ± 0.5g	0.031	[68]
		CC	duration of prednisone treatment in PMR patients		41.8 ± 39.3 vs. 16.0 ± 10.4 months	0.10	
		CC	a number of relapses in PMR patients		0.9 ± 1.3 vs. 0.0 ± 0.0	0.07	
CCR5Δ32	88 PMR patients 86 controls Italian patients (Reggio Emilia)	CCR5 or CCR5Δ32	initial and cumulative dose of prednisone, duration of therapy, relapse/recurrence in PMR patients vs. healthy controls			NS	[69]
IL-1RN 86 bp VNTRs within the second intron	139 iPMR patients 437 controls Spanish patients (Cantabria region)	*1 (four repeats) or *2 (two repeats)	relapses/recurrences, number of relapses, duration of corticosteroid therapy, cumulative prednisone dose			NS	[56]
IL-1A +4845 C/T	92 PMR patients 79 controls Italian patients (Reggio Emilia)	C or T	relapses/recurrences, duration of corticosteroid therapy, cumulative prednisone dose			NS	[70]
IL-1B −511		*1 or *2				NS	
IL-1B +3954		*1 or *2				NS	
TNFA −308		*1 or *2				NS	
IL-1RN Intron 2		*1 or *2				NS	
ICAM-1 241 R/G	72 iPMR patients Spanish patients (Lugo region)	R or G	patients with vs. without relapse			NS	[71]
ICAM-1 469 K/E		K or E	patients with vs. without relapse			NS	
HLA-DRB1		*0401	patients with vs. without relapse		7.2 (1.5–35.5)	0.01	
HLA-DRB1 and ICAM-1 241		*0401 and GG	patients with vs. without relapse		15.2 (2.3–99.5)	0.005 0.03 ^#^	
ICAM-1 241 R/G	91 iPMR patients 228 controls Italian patients (Reggio Emilia)	RR + GR vs. GG	relapses/recurrences		1.6 (1.1–2.4)	0.01	[72]
IL-10 −592C/A	168 iPMR patients 124 controls Spanish patients (Cantabria region)	C or A	relapses/recurrences, number of relapses, duration of corticosteroid therapy, cumulative prednisone dose			NS	[73]
IL-10 −1082A/G		A or G				NS	
NOS3 −786T/C	78 iPMR patients 2061 controls German patients (Heidelberg)	T or C	steroid responsiveness			NS	[74]

CCR5, CC chemokine receptor 5; HLA, Human leukocyte antigen; ICAM-1, Intercellular adhesion molecule-1; IL, Interleukin; IL-1Ra, IL-1 receptor antagonist; iPMR, Isolated PMR; NOS3, Nitric oxide synthase 3; TLR, Toll-like receptor; TNFA, Tumor necrosis factor α. NS—no statistical differences. ^#^ values corrected by the number of comparisons.

## Abbreviations

AP1	Activator protein 1
CCR5	CC chemokine receptor 5
BANK1	B cell scaffold protein with ankyrin repeats 1
BLK	B-lymphoid kinase
CRH	Corticotropin-releasing hormone
CRP	C-reactive protein
ERK	Extracellular signal-regulated kinase
ESR	Erythrocyte sedimentation rate
GCA	Giant cell arteritis
GCs	Glucocorticoids
GPIIIa	platelet glycoprotein IIIa
GR	Glucocorticoid receptor
HLA	Human leukocyte antigen
ICAM-1	Intercellular adhesion molecule-1
IFN-γ	Interferon gamma
INFG	IFN-γ gene
IL	Interleukin
IL-1Ra	IL-1 receptor antagonist
IRF	IFN regulatory factor
ITGAM	Integrin alpha M
IκBα	Inhibitor kappa B alpha
JNK	c-Jun N-terminal kinase
MAP	Mitogen-activated protein
MCP-1	Monocyte chemoattractant protein-1
MHC	Major histocompatibility complex
MIF	Macrophage migration inhibitory factor
MPO	myeloperoxidase
NFκB1	Nuclear factor of κ-light polypeptide gene enhancer in B cells 1
PET	positron emission tomography
PMR	Polymyalgia rheumatica
PTPN22	Protein tyrosine phosphatase non-receptor 22
RA	rheumatoid arthritis
RANTES	Regulated upon activation, normal T cell expressed and presumably secreted
SNP	Single nucleotide polymorphism
STAT	Signal transduction-activated transcription factor
TLR	Toll-like receptor
TNF	Tumor necrosis factor
TNFAIP3	AIP3, Tumor necrosis factor-α-induced protein 3
TRAF	TNF receptor-associated factor
VNTRs	Variable number of tandem-repeats
VEGF	Vascular endothelial growth factor
